# HRVCam: robust camera-based measurement of heart rate variability

**DOI:** 10.1117/1.JBO.26.2.022707

**Published:** 2021-02-10

**Authors:** Amruta Pai, Ashok Veeraraghavan, Ashutosh Sabharwal

**Affiliations:** Rice University, Scalable Health Labs, Electrical and Computer Engineering Department, Houston, Texas, United States

**Keywords:** heart rate variability, imaging photoplethysmography, noncontact HRV, pulse frequency demodulation

## Abstract

**Significance**: Non-contact, camera-based heart rate variability estimation is desirable in numerous applications, including medical, automotive, and entertainment. Unfortunately, camera-based HRV accuracy and reliability suffer due to two challenges: (a) darker skin tones result in lower SNR and (b) relative motion induces measurement artifacts.

**Aim**: We propose an algorithm HRVCam that provides sufficient robustness to low SNR and motion-induced artifacts commonly present in imaging photoplethysmography (iPPG) signals.

**Approach**: HRVCam computes camera-based HRV from the instantaneous frequency of the iPPG signal. HRVCam uses automatic adaptive bandwidth filtering along with discrete energy separation to estimate the instantaneous frequency. The parameters of HRVCam use the observed characteristics of HRV and iPPG signals.

**Results**: We capture a new dataset containing 16 participants with diverse skin tones. We demonstrate that HRVCam reduces the error in camera-based HRV metrics significantly (more than 50% reduction) for videos with dark skin and face motion.

**Conclusion**: HRVCam can be used on top of iPPG estimation algorithms to provide robust HRV measurements making camera-based HRV practical.

## 1 Introduction

The nervous and the cardiac systems in the human body are intimately connected, primarily through the autonomous nervous system. This dynamic interplay is reflected in the beat-to-beat variation of the heart rate, formally labeled as heart rate variability (HRV). Interbeat interval (IBI) quantifies the time period between consecutive heartbeats. Several quantitative HRV metrics such as root mean square of successive differences in interbeat intervals (RMSSD) and standard deviation of interbeat intervals (SDNN) summarize the changes in the IBIs.^1^^,^^2^

HRV is clinically relevant because it provides a surrogate measure of the health of the autonomous nervous system. A low-baseline HRV is a symptom of poor autonomic function seen in diseases such as sudden cardiac death^3^ and diabetic autonomic neuropathy.^4^ Normal values of short term HRV metrics are 32 to 93 ms for SDNN and 19 to 75 ms for RMSSD.^5^

HRV is clinically measured using electrocardiography (ECG)^6^ with well-defined controlled protocols. However, ECG can be limiting because the electrical leads need to be in contact with the skin surface. Contact may not always be feasible for applications such as driver stress detection, behavioral sensing, and monitoring for symptoms of sudden cardiac death in neonatal care units. Thus, many applications would benefit if robust camera-based HRV measurement were available.

Noncontact measurement of HRV may be possible with camera-based imaging photoplethysmography (iPPG)^7^^,^^8^ due to two factors. First, the optical photoplethysmography (PPG) signal enables the measurement of pulse rate variability. Pulse rate variability is shown to be correlated to HRV.^9^ Second, the PPG signal can be captured by the camera placed at a distance from the participant.

Noncontact HRV estimation suffers from several disadvantages compared to contact HRV estimation. First, camera-based methods result in low SNR due to the absorption of incident light by high amounts of melanin pigment in dark skin tissue. Second, camera-based noncontact methods have to contend with unpredictable illumination changes due to relative nonrigid movements of the skin surface. The unpredictable illumination changes corrupt the shape of the iPPG signal rendering crucial IBIs not easily measurable. The disadvantages are prominent in iPPG signals because both the light source (e.g., ambient light) and light detector (i.e., the camera) are at a distance from the skin surface.

A standard time-based method to measure HRV is to detect peaks in the PPG or ECG signal and then estimate HRV from the measured time differences of the occurrence of the peaks. However, peak-based approaches typically perform poorly due to the low SNR and often high-motion-related artifacts in iPPG signals.

An alternate approach to peak-based estimation is to measure HRV using pulse frequency demodulation (PFDM)^10^^,^^11^ that relies on the instantaneous frequency. Chou et al.^12^ demonstrated that the frequency demodulation approach was more robust than the peak selection method for noisy contact photoplethysmography (cPPG) signals.

We investigated the use of PFDM to improve the accuracy of HRV metrics measured from a low signal quality iPPG signal. The main contributions of this paper are twofold:

• *HRVCam algorithm*. We propose HRVCam, a new algorithm based on a frequency demodulation framework to estimate the instantaneous frequency of the iPPG signal. The framework is a combination of a new automated adaptive bandpass filter and the discrete energy separation algorithm (DESA).^13^
• *HRVCam dataset*. We collected a new iPPG dataset with validated ground truth using a pulse oximeter under different scenarios: (i) low melanin pigment (light skin tones), (ii) high melanin pigment (dark skin tones), (iii) low motion, such as sitting still, and (iv) different degrees of natural motion (reading, watching, and talking). The new dataset is publicly available. Evaluation of HRVCam on the dataset shows improved performance of HRVCam when compared to existing state-of-the-art approaches.

### 1.1 Prior Work

#### 1.1.1 Contact-based HRV measurement

The most prevalent algorithms to measure contact-based HRV from ECG or cPPG signals are time-based peak detection algorithms with artifact removal filters such as the noncausal variable threshold (NC-VT) filter.^14^ The NC-VT filter removes incorrect values based on the local statistics of the IBI time series.

An alternative to direct time-based peak detection approaches is utilizing the frequency characteristics of the cPPG signal. PFDM^10^^–^^12^ estimates IBIs from the instantaneous frequency of the cPPG signal. There are two approaches to PFDM. The first approach is the complex demodulation (CDM) of the cPPG signal.^10^^,^^11^ The second approach consists of extracting the fundamental component of the cPPG signal and performing Hilbert transformation to estimate the instantaneous frequency.^12^ PFDM approaches are less sensitive than peak detection approaches to the sensor noise in the cPPG signal.^12^

Although contact-based HRV is the clinical gold-standard, it is impractical for many emerging applications. In applications such as neonatal intensive care units, noncontact HRV measurements are the only practical alternative. Noncontact HRV measurements are possible with camera-based iPPG systems. However, the algorithms designed for HRV estimation from contact cPPG signals are not as effective for noncontact iPPG signals. Traditionally, HRV algorithms have been designed for cPPG signals collected under restrictive ideal conditions, with the primary source of noise being sensor noise and power line interference noise. However, the primary source of noise in iPPG signals is motion artifacts. Algorithms designed to handle sensor noise and power line interference cannot handle motion artifacts.

#### 1.1.2 Imaging photoplethysmography

Over the past few years, camera-based iPPG system^8^^,^^15^^,^^16^ has received significant attention for its potential for noncontact heart rate and HRV measurement. However, the iPPG signal presents a tough challenge for heart rate and HRV measurement because of its low signal quality. Most of the early works^8^^,^^15^^,^^16^ focused on improving the signal quality of iPPG and heart rate measurement.

Independent component analysis^8^ applied to intensities measured from the red, green, and blue channels improved the quality of the iPPG signal. Chrominance^15^-based iPPG signal estimation allowed robust heart rate measurements in high-motion scenarios. The distancePPG^16^ algorithm maximized the overall SNR of the iPPG signal using a maximal ratio combination with a goodness metric calculated over smaller pixel regions.

#### 1.1.3 Noncontact measurement of HRV

Recently, there is emerging interest in the potential for HRV measurement from iPPG signals.^7^^,^^16^^–^^18^ Most of the related efforts in this direction^7^^,^^16^^–^^18^ adopted custom peak detection algorithms inspired by time-based peak detection for HRV measurement from ECG signals. A peak detection-based algorithm performs poorly in low SNR iPPG signals because it gives rise to false positives that adversely affect HRV estimation.^16^

Previous works^7^^,^^19^ used custom approaches to filter false positives and reduce errors in HRV measurements. One of the methods^7^ included a semiautomated procedure with manual validation and an IBI threshold to remove false positives. Poh et al.^19^ used the NC-VT algorithm to filter noisy IBIs.

Most iPPG HRV algorithms function in the time domain of the iPPG signal. PFDM methods^10^^–^^12^ that are shown to be more robust than time-based approaches for cPPG signals have not been employed with iPPG signals. In the proposed algorithm HRVCam, we adopt a PFDM scheme to measure HRV by mainly using the frequency characteristics of the iPPG signal.

In previous iPPG HRV works,^7^^,^^19^ the accuracies of HRV parameters were reported for low-motion scenarios. Most of the past work^7^^,^^17^^,^^18^ lacked an extensive analysis of HRV metrics’ accuracies for different skin tones and varying degrees of motion. We evaluate the HRVCam algorithm across diverse skin tones and motion scenarios to provide an extensive analysis of noncontact camera-based HRV measurements.

## 2 Methods

### 2.1 Background and Model

In a camera-based HRV system, the exposed skin tissue is recorded by the camera while the participant is performing an activity facing the camera. The camera captures the light reflected from the exposed skin tissue. The captured intensity contains subtle intensity variations over time. The subtle temporal intensity variations arise due to the temporal changes in blood volume flowing in the microvasculature beneath the exposed skin tissue. The iPPG signal is a noisy estimate of this tiny intensity variation. The objective is to estimate HRV metrics from the iPPG signal computed from the exposed skin tissue video. First, we define the HRV metrics. Next, we define the signal model assumed for the iPPG signal computed from the video.

#### 2.1.1 HRV metrics

HRV is the variation in IBI. The IBI of the n’th heartbeat is defined as

$$\mathrm{IBI}(n)={\text{time}}_{\text{peak}}(n)-{\text{time}}_{\text{peak}}(n-1),$$ (1)

where ${\text{time}}_{\text{peak}}(n)$ is the timing of the peak corresponding to n’th heartbeat in the PPG signal p(t). The variation in IBI is summarized by various statistical metrics and spectral metrics.^1^ Commonly used statistical metrics for representing short-time HRV (over a time duration of the order of 10 s to 1 min) are SDNN and RMSSD. SDNN is defined as

SDNN=σ(IBI), (2)

where σ is the standard deviation. SDNN captures the low-frequency variation in IBI. RMSSD is root mean square of successive differences of IBIs. RMSSD is related to the high-frequency variation of IBI

$$\mathrm{RMSSD}=\sqrt{\frac{\sum {\left[\mathrm{IBI}(n)-\mathrm{IBI}(n-1)\right]}^{2}}{N-1},}$$ (3)

where N is the number of heartbeats detected in a considered time duration, commonly chosen to be 60 s. The RMSSD is the primary time-domain measure used to estimate the vagally mediated changes reflected in HRV.^20^

#### 2.1.2 Frequency modulated iPPG signal model

The overall iPPG signal i(t) computed from the video is represented in terms of the following components shown in Eq. (4). The PPG signal p(t) arises from subtle intensity variation due to subsurface reflection of incident light by chromophores present in blood vessels beneath the skin surface. PPG strength α denotes the signal strength of the subsurface reflection due to pulsatile blood volume changes. PPG strength α depends on blood perfusion of the skin surface and melanin content of the skin. The melanin pigment (higher in darker skin tones) absorbs the incident light, resulting in lower modulation of the incident light by the pulsatile blood volume change. Thus α is lower for dark skin tones. The motion interference n(t) is the noise signal that arises due to complex interaction between the incident light and skin tissue during facial motion or head movements. The surface reflectance b is constant. Finally, q(t) is the camera quantization noise because the strength of the PPG signal is minimal compared to the large surface reflectance b

i(t)=αp(t)+b+n(t)+q(t). (4)

The PPG signal p(t) is an amplitude-modulated-frequency-modulated (AM-FM) signal.^10^ We propose to model p(t) as a quasi-periodic signal given by

$$p(t)=\sum a_{k}(t)\cos[\phi_{k}(t)].$$ (5)

The instantaneous frequency of the PPG signal p(t) captures the HRV.^11^ The instantaneous frequency f(t) is the derivative of the instantaneous phase ϕ(t) of the signal. The instantaneous frequency $f_{k}(t)$ for the k’th harmonic is then given as

$$f_{k}(t)=\frac{1}{2\pi }\frac{d\phi_{k}(t)}{dt},$$ (6)

$$f_{k}(t)=k[f_{\mathrm{hr}}+f_{\mathrm{hrv}}(t)],$$ (7)

where $f_{\mathrm{hr}}$ is the mean heart rate frequency and $f_{\mathrm{hrv}}(t)$ is the change in instantaneous frequency due to HRV.

The PPG signal p(t) stated in Eq. (5) consists of multiple harmonics. It is well known that the energy of the iPPG signal i(t) is largely concentrated in the first harmonic of the PPG signal p(t).^16^ We propose that the first harmonic signal of p(t) be modeled as the following equations:

$$p_{h1}(t)=a(t)\cos\{2\pi \int [f(t)]dt\},$$ (8)

$$p_{h1}(t)=a(t)\cos\{2\pi \int [f_{\mathrm{hr}}+f_{\mathrm{hrv}}(t)]dt\}.$$ (9)

The signal $p_{h1}(t)$ is parameterized as a(t) and f(t) that correspond to amplitude modulation and frequency modulation, respectively.^21^ As previously mentioned, the frequency modulation arises due to the influence of the autonomous nervous system.

The first harmonic $p_{h1}(t)$ can be compared to a frequency modulated (FM) signal s(t) shown in the following equation

$$s(t)=a\;\cos\{2\pi \int [f_{c}+\Delta_{f}x_{m}(t)]dt\},$$ (10)

where $f_{\mathrm{hr}}$ is equivalent to the carrier frequency $f_{c}$ and $f_{\mathrm{hrv}}(t)$ is equivalent to the modulating signal $\Delta_{f}x_{m}(t)$. $\Delta_{f}$ is the frequency deviation.

The signal model of the iPPG signal i(t) in terms of the HRV information $f_{\mathrm{hrv}}(t)$ and different noises n(t) and q(t) is as follows:

$$i(t)=\alpha a(t)\cos\{2\pi \int [f_{\mathrm{hr}}+f_{\mathrm{hrv}}(t)]dt\}+b+n(t)+q(t).$$ (11)

### 2.2 Challenge of Camera-Based HRV Estimation

The primary aim is that given the iPPG signal i(t), we want to robustly extract the subtle HRV information from the PPG signal p(t). However, i(t) is corrupted with different noise sources.

The signal strength α of the PPG signal p(t) is weak compared to the surface reflection b captured by the camera. As a result, the quantization error q(t) influences the quality of the PPG signal p(t). The signal strength is lower for darker skin tones due to high melanin pigment in the exposed skin surface. Thus, the quantization error q(t) is a more prominent source of noise in the computed iPPG signals i(t) for darker skin tissue.

**Fig. 1:**
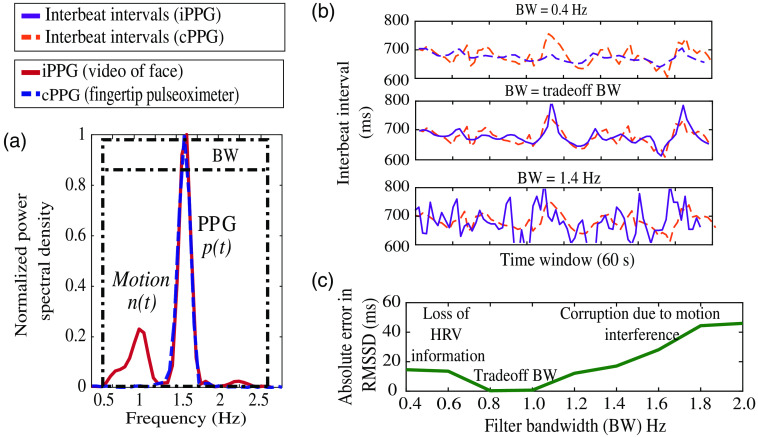
(a) Comparison of the normalized power spectral density of the video iPPG signal and the ground truth cPPG signal. (b) IBI estimates from iPPG compared with the ground truth for different BWs. The estimates are most accurate for the BW of 1 Hz (middle figure). (c) Trade-off between losing HRV information and rejecting motion interference by comparing the error in RMSSD estimate across different BWs.

In scenarios with head movements or facial motion, the motion interference signal n(t) overlaps with the PPG signal p(t) in the time domain. The power spectral density of a 10-s epoch of the iPPG signal i(t) is shown in Fig. 1(a). In the frequency domain, the motion interference signal is observed as spurious frequency components shown in Fig. 1(a). To compute the IBI of the PPG signal p(t), we filter the i(t) with some bandwidth (BW) around the fundamental heart rate frequency. However, the BW needed to filter the iPPG signal i(t) is not trivial. A wider BW of say 2 Hz (0.5 to 2.5 Hz) that is mostly used in the prior work^16^^,^^17^ does not reject n(t). Thus, the IBI estimated is highly erroneous compared to the ground truth, as seen in Fig. 1(b). If we choose a narrow BW of 0.4 Hz, we risk losing HRV information and obtain smoothed estimates of IBI as seen in Fig. 1(b). We calculate the error in estimated RMSSD after filtering with different BWs. The error is calculated against RMSSD derived from the IBI computed from the ground truth pulse oximeter signal. Hence, it is evident in Fig. 1(c) that there exists a BW that is a sweet spot in terms of retaining HRV information and rejecting motion interference. Let us call this sweet spot BW as the trade-off BW. For the example shown in Fig. 1(a), the trade-off BW is a BW of 1 Hz. The IBI of all epochs after filtering with the trade-off BW computed for each 10-s epoch follows the ground truth more closely, as seen in Fig. 1(b). The trade-off BW differs for each epoch of the iPPG signal as it depends on the motion interference present in that epoch.

The HRV estimate is very sensitive to the BW. Since the HRV information is associated with the quasi-periodicity of the PPG signal, the HRV information is present in the sidebands of the fundamental harmonic $p_{h1}(t)$. Thus, the bandpass filter needs to have the center frequency equal to the heart rate frequency and BW that retains the desired HRV information.

**Fig. 2:**
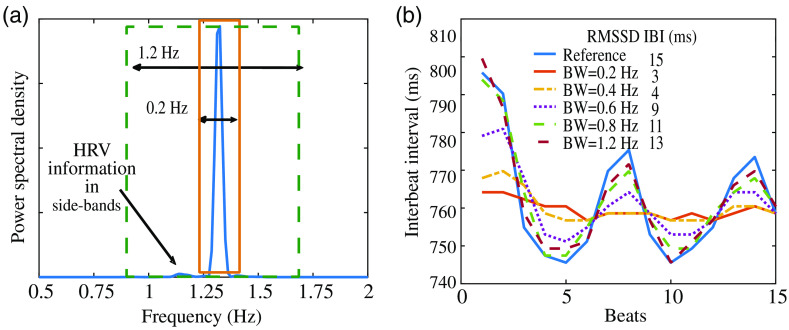
(b) On using a large BW and including the information in the sidebands of the fundamental harmonic, the estimates of HRV RMSSD are more accurate and follow ground truth more closely. (a) HRV information is in the sidebands of the fundamental harmonic because of quasi-periodicity of pulse signal.

To demonstrate the effect of the filter BW on the HRV parameter RMSSD further, we bandpass a clean cPPG signal with different filter BWs ranging from 0.2 to 1.2 as shown in Fig. 2(a). We then compute the RMSSD HRV parameter from the IBI of the filtered first harmonic signal. In Fig. 2(b), we observe that the bandpass filter BW that controls the retention of the sideband signal information has a direct effect on the measurement of the RMSSD HRV parameter. When the BW is narrow, the RMSSD metric computed is less accurate. The error occurs because we lose the HRV information present in the sidebands of the fundamental frequency. The loss in sideband HRV information leads to smoothed out estimates of the IBI, as shown in Fig. 2(b). In contrast, we retain more of the HRV information on increasing the BW. As a result, the computed IBI is more comparable to the reference ground truth IBI measured from the unfiltered cPPG signal.

Thus, filtering the first harmonic of the iPPG signal with a bandpass filer is not trivial. Using a wideband bandpass filter for the iPPG signal ensures high retention of sideband HRV information, but also leads to incomplete removal of motion interference. Removing the motion interference but not filtering out the subtle HRV information is a major challenge for estimating IBI from the measured intensity signal i(t). We tackle this challenge with the proposed algorithm HRVCam.

### 2.3 HRVCam: Algorithm Description

In this section, we propose the HRVCam algorithm as an estimator to extract IBI from the iPPG signal i(t) computed from videos of exposed skin tissue. We used the chrominance-based remote-PPG (rPPG)^15^ and distancePPG^16^ algorithm to compute the iPPG signal i(t) from the videos of the exposed skin surface. We estimated $f^{n}(t)$ for every epoch of time duration T. The underlying assumption is that the PPG signal $p^{n}(t)$ present in i(t) is quasi-periodic in the epoch n. Thus, in a given epoch n, $f_{\mathrm{hrv}}^{n}(t)\ll f_{\mathrm{hr}}^{n}$ by the definition of quasi-periodicity.

#### 2.3.1 Estimation of HRV in high SNR regimes

In high SNR regimes, the IBIs of the PPG signal p(t) can be estimated by a time-based peak detection approach. The fiducial points of the PPG signal waveform corresponding to every heartbeat are detected. The time difference between the fiducial points is calculated to find IBI. However, in low SNR regimes (as expected in camera-based measurements), the peak locations in the iPPG signal i(t) are significantly corrupted. As the peak times do not capture the frequency content specific to p(t), relying on the peak timings alone provides a noisy estimate of IBI. Hence, we need to find estimators that are robust in the presence of noise to measure HRV from iPPG signals i(t).

#### 2.3.2 Estimation of HRV in low SNR regimes

Quantization error can be modeled as additive white noise.^22^ Chou et al.^12^ showed that capturing the instantaneous frequency as a measure of HRV is more robust than peak detection in the presence of additive white noise. We used frequency demodulators to extract the instantaneous frequency of the PPG signal, specifically for camera-based HRV systems. In HRVCam, we propose a PFDM method (PFDM) to estimate the instantaneous frequency $f^{n}(t)$ of the fundamental harmonic signal $p_{h1}^{n}(t)$ of the n’th epoch with time duration T. The proposed PFDM method is inspired from PFDM algorithms^10^^–^^12^ designed for the cPPG signals.

Let FM signal $p_{h1}^{n}(t)$ have a bandwidth (BW) given as BW. The BW is driven by the HRV information present in the sidebands of the fundamental harmonic. A bandpass filter with central frequency $f_{\mathrm{hr}}^{n}$, low- and high-frequency cut-offs as $f_{\mathrm{hr}}^{n}-\frac{\mathrm{BW}}{2}$ and $f_{\mathrm{hr}}^{n}+\frac{\mathrm{BW}}{2}$, respectively, is used to separate $p_{h1}^{n}(t)$ from $p^{n}(t)$.

We extracted the instantaneous frequency $f^{n}(t)$ after extracting the first harmonic signal $p_{h1}^{n}(t)$. The extraction of instantaneous frequency is a typical frequency demodulation problem. There are many frequency demodulation algorithms developed by the communications theory community. Previous work^12^ used the Hilbert transform method for the extraction of instantaneous frequency from the fundamental harmonic signal. The Hilbert transform approach was not suitable in our application as the short-time windows may be insufficient for a good estimation of the instantaneous frequency.^23^ Instead, we chose to utilize the DESA that is not dependent on multiple time periods for accurate estimation of instantaneous frequency.^13^ The DESA algorithm is also computationally less expensive than the Hilbert transform method.^23^ DESA takes as input the first harmonic signal $p_{h1}^{n}(t)$ and provides the instantaneous frequency $f^{n}(t)$ as output.

The signal $p_{h1}^{n}(t)$ can be represented as a discrete sequence x(m), where m corresponds to discrete samples of the signal. Equations (1214) show the steps of the DESA algorithm

$$x(m)=p_{h1}^{n}(t),\;y(m)=x(m)-x(m-1),\;\psi [x(m)]=x^{2}(m)-x(m+1)x(m-1).$$ (12)

We filtered ψ[x(m)] to remove higher order components that may arise due to the $x^{2}(m)$

$$G(m)=1-\frac{\psi [y(m)]-\psi [y(m+1)]}{4\psi [x(m)]},$$ (13)

$$\omega (m)=\{\begin{array}{ll} \arccos[G(m)] & \text{if }\cos[2\pi (0.5f_{\mathrm{hr}}^{n})]<|G(m)|<\cos[2\pi (1.5f_{\mathrm{hr}}^{n})]\\ 2\pi f_{\mathrm{hr}}^{n} & \text{otherwise} \end{array}.$$ (14)

To avoid spurious estimates that arise due to abrupt discontinuities in the signal from sudden sparse noise artifacts, we assigned a threshold to G(m) while computing ω(m) shown in Eq. (14).

We filtered ω(m) using a low-pass filter of order 200 with a cut-off of 0.6 Hz to remove any leaked energy beyond the spectral bandwidth of the HRV signal. The spectral bandwidth is <0.5  Hz.^20^ Next, we calculated the final instantaneous frequency estimate as shown in the following equation:

$$f^{n}(t)=\frac{\omega (m)}{2\pi }.$$ (15)

The final f(t) is achieved by stitching together $f^{n}(t)$. To calculate HRV statistical metrics, we needed IBIs (time difference between consecutive beats). We computed the beat timings and corresponding IBIs from the reconstructed FM signal r(t). The signal r(t) was reconstructed from the extracted denoised instantaneous frequency f(t) as shown in Eq. (16). The reconstructed FM signal r(t) was interpolated to 500 Hz using spline interpolation to improve the temporal resolution of the beat timings

r(t)=cos[∫2πf(t)dt]. (16)

#### 2.3.3 Estimation of HRV in the presence of motion interference

Motion interference noise n(t) is a structured source of the noise. The magnitude and frequency of n(t) depend entirely on the subject’s specific movement in the video. The motion interference signal n(t) overlaps with the signal of interest p(t) in the time domain, which renders peak detection methods ineffectual for HRV estimation. If the signal n(t) is sparse, the inaccurate beats can be filtered out by algorithms such as the NC-VT algorithm. However, when n(t) is nonsparse or periodic, the IBI are skewed and not reflective of the true frequency variation of the PPG signal p(t).

The performance of prior PFDM techniques^11^^,^^12^ has not been evaluated for cPPG signals with noise resembling the motion interference signal. In theory, our proposed PFDM method described in Sec. 2.3.2 would be effective if the signal n(t) is completely filtered out while extracting the first harmonic signal $p_{h1}^{n}(t)$.

The peak detection method on the signal i(t) shown in Fig. 1(a) gives an estimation error of 30 ms. Using the proposed PFDM approach described in Sec. 2.3.2, the estimation error reduces to 15 ms. The estimation error is still high as the frequency components of the signal n(t) are random and may be present within the range of the fixed filter BW used to extract the first harmonic signal of the PPG signal. To minimize the effect of the interference, we propose to introduce an adaptive bandwidth (aBW) for the bandpass filter. The filter’s aBW is automatically estimated from signal i(t) itself.

Let us denote the signal i(t) in a single epoch T as $i^{n}(t)$. The power spectrum of $i^{n}(t)$ is $I^{n}(f)$. We assumed that since the subject is at rest, the average heart rate $f_{\mathrm{hr}}^{n}$ does not change more than $\tau_{\mathrm{hr}}$ between consecutive epochs. The fundamental dominant frequency given by $f_{\mathrm{hr}}^{n}$ is the average heart rate frequency for the n’th epoch. $f_{\mathrm{hr}}^{\text{avg}}$ is the frequency corresponding to is the peak of power spectrum (Hanning window) I(f). $f_{\mathrm{hr}}^{n}$ is computed by calculating the peak of the power density spectrum $I^{n}(f)$. We designed the bandpass filter with the following frequencies, the center frequency $f_{c}^{n}$, lower cut-off frequency $f_{l}^{n}$, and higher cut-off frequency $f_{h}^{n}$. The frequencies for the bandpass filter in the n’th epoch are calculated as shown in the following equations:

$$f_{c}^{n}=\{\begin{array}{ll} f_{\mathrm{hr}}^{n} & \text{if }|f_{\mathrm{hr}}^{n}-f_{\mathrm{hr}}^{n-1}|<\tau_{\mathrm{hr}}\text{ and }|f_{\mathrm{hr}}^{n}-f_{\mathrm{hr}}^{\text{avg}}|<\tau_{\mathrm{hr}},\\ f_{\mathrm{hr}}^{n-1} & \text{otherwise.} \end{array}\;$$ (17)

$$f_{l}^{n}=\{\begin{array}{ll} f_{c}^{n}-\frac{\mathrm{BW}}{2} & \text{if }I^{n}(f)<\tau_{p}I^{n}(f_{c}^{n})\text{ for }f\in (f_{c}^{n}-\frac{\mathrm{BW}}{2},f_{c}^{n}-0.3)\\ \max(f) & \text{if }I^{n}(f)>\tau_{p}I^{n}(f_{c}^{n})\text{ for }f\in (f_{c}^{n}-\frac{\mathrm{BW}}{2},f_{c}^{n}-0.3) \end{array},$$ (18)

$$f_{h}^{n}=\{\begin{array}{ll} f_{c}^{n}+\frac{\mathrm{BW}}{2} & \text{if }I^{n}(f)<\tau_{p}I^{n}(f_{c}^{n})\text{ for }f\in (f_{c}^{n}+0.3,f_{c}^{n}+\frac{\mathrm{BW}}{2})\\ \min(f) & \text{if }I^{n}(f)>\tau_{p}I^{n}(f_{c}^{n})\text{ for }f\in (f_{c}^{n}+0.3,f_{c}^{n}+\frac{\mathrm{BW}}{2}) \end{array}.$$ (19)

The adaptive filter with bandwidth $\text{aBW=}f_{h}^{n}-f_{l}^{n}$, selects cut-off frequencies around $f_{\mathrm{hr}}^{n}$ such that the power density at these cut-off frequencies is much less compared to the power at the fundamental heart rate frequency. If sideband frequencies upto $f_{c}^{n}-\frac{\mathrm{BW}}{2}$ have a power spectral density $P_{i}^{n}(f)$ less than $\tau_{p}$ times the power spectral density at $f_{c}^{n}$, the power in the sideband is associated with HRV information. Hence, we included the frequencies in the passband of our filter. When motion interference is present in the sideband, we selected the lower filter cutoff $f_{l}^{n}$ to be the closest frequency to $f_{c}^{n}$ that has a power spectral density greater than $\tau_{p}$ times the power spectral density at $f_{c}^{n}$. We used a similar strategy for higher filter cutoff. In the first epoch, $f_{c}^{n}=f_{\mathrm{hr}}^{\text{avg}}$ if condition in Eq. (17) is not satisfied.

To prevent abrupt changes in the cut-off frequencies of the bandpass filter from successive epochs, the cut-off frequencies used in the bandpass filter $f_{l}^{n}$ and $f_{h}^{n}$ are smoothed using cut-off frequencies from previous window as shown in the following equation:

$$f_{l}^{n}=\frac{f_{l}^{n}+f_{l}^{n-1}}{2},\;f_{h}^{n}=\frac{f_{h}^{n}+f_{h}^{n-1}}{2}.$$ (20)

Thus, with a 200 order finite impulse response (FIR) bandpass filter with cut-off frequencies $f_{l}^{n}$ and $f_{h}^{n}$, the first harmonic signal $p_{h1}^{n}(t)$ in an epoch is selected.

After selecting the first harmonic signal, DESA shown in Eqs. (12)–(14) was used to extract the instantaneous frequency.

#### 2.3.4 Implementation of HRVCam

**Fig. 3:**
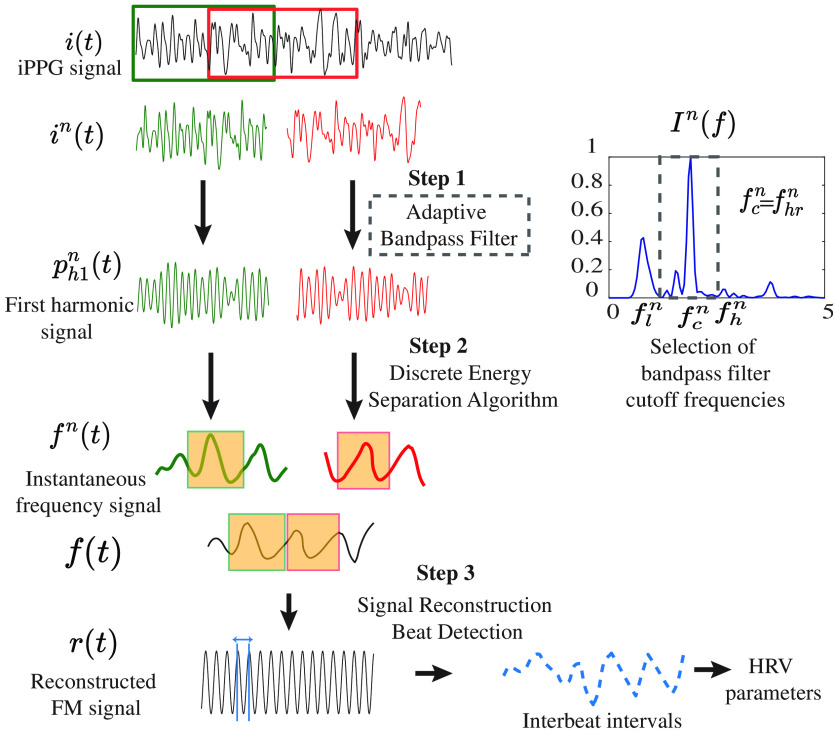
Flowchart of HRVCam: iPPG signals extracted from raw videos is divided into epochs. Step 1: iPPG signal passed through an adaptive bandpass filter that estimates the cut-off frequencies of the filter based on the noise structure in the iPPG signal. The bandpass filter filters out the fundamental harmonic signal. Step 2: Discrete energy separation algorithm was used to extract out the instantaneous frequency. Step 3: the instantaneous frequency is converted to interbeat intervals by reconstructing the FM signal.

Figure 3 depicts the flowchart of the proposed algorithm HRVCam. After deriving the iPPG signal from the videos using any off the shelf iPPG algorithm, HRVCam estimates HRV from the noisy iPPG signal i(t). The signal i(t) is divided into multiple epochs $i^{n}(t)$. We chose a sliding window of 10 s with a sliding interval of 1-5 s. A smaller sliding interval was chosen for motion scenarios. The sampling frequency is 30 Hz. HRVCam computed $f^{n}(t)$ for every epoch n. In step 1, the fundamental harmonic $p_{h1}^{n}(t)$ was extracted with the adaptive bandpass filtering approach as explained in Eqs. (17)–(20). In step 2, DESA shown in Eqs. (12)–(15) was used to extract the instantaneous frequency $f^{n}(t)$ from the fundamental harmonic. We obtained the final instantaneous frequency f(t) of the signal i(t) by concatenating the signal between 2.5 and 7.5 s of $f^{n}(t)$ over multiple epochs. In the step 3, the instantaneous frequency was then converted to IBIs by reconstructing with a cosine function as demonstrated Eq. (16).

Three main parameters are used in the algorithm.

1. BW: the BW of the FM fundamental harmonic signal $p_{h1}(t)$.
2. $\tau_{p}$: relative threshold for sideband power of the fundamental harmonic $p_{h1}(t)$.
3. $\tau_{\mathrm{hr}}$: maximum difference between fundamental frequency between any consecutive epoch.

As previously mentioned, we modeled the fundamental harmonic signal $p_{h1}(t)$ as an FM signal s(t) as shown in Eqs. (8) and (10). Based on the frequency modulation model, we provided suitable values for parameters BW and $\tau_{p}$. The change in IBI is small, of the order of 32 to 93 ms^20^ that, in terms of frequency deviation $\Delta_{f}$, is approximately 0.2 Hz. The power spectrum of the HRV signal, which is constructed by a sequence of IBI from multiple epochs, has a BW of <0.5. Hz.^20^ Thus using the Carson’s rule, the BW of interest around the central frequency ($f_{\mathrm{hr}}$) that contains HRV information was derived as follows:

$$\mathrm{BW}=2(\Delta_{f}+f_{m})=2(0.2+0.5)=1.4\;\mathrm{Hz}.$$ (21)

Based on the frequency modulation model, we also calculate the modulation index $m=\Delta f/f_{m}$. On calculation, m equals 0.4<1, which corresponds to narrowband frequency modulation. Thus, most of the energy is concentrated in the BW of 1.4 Hz around the central frequency of $f_{\mathrm{hr}}$. For m<1, over 80% of the energy will be present at the carrier frequency ($f_{\mathrm{hr}}$), which is the fundamental frequency.^24^ The sidebands frequencies that lie in the range $\left(f_{\mathrm{hr}}-\frac{\mathrm{BW}}{2},f_{\mathrm{hr}}+\frac{\mathrm{BW}}{2}\right)$ have an energy of <20% of the total energy. Based on this analysis, we note that if the sideband power amplitude is >20% of the power amplitude at the central frequency, the source of energy in the sideband frequencies is due to motion interference rather than HRV information. For the epochs where the sideband power amplitude is >20%, we used a narrow bandpass filter to remove motion interference in the sidebands. Otherwise, we used a wide bandpass filter to include all the HRV information within that epoch. Hence, we used a value of 0.2 for the parameter $\tau_{p}$. The parameter $\tau_{\mathrm{hr}}$ was set to a value of 0.4.

## 3 Dataset

We collected a dataset of iPPG signals from participants with diverse skin tones and natural motions to evaluate our algorithm.

### 3.1 Data Collection

**Fig. 4:**
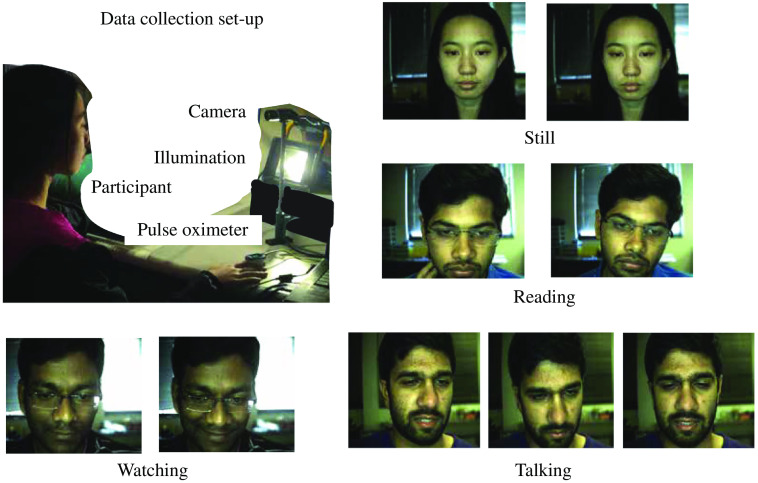
Typical face video-based iPPG signal acquisition system. Participants performed different tasks such as sitting still, reading a facts page, watching a scary movie trailer, and talking about a personal experience in front of the camera.

We had 16 individuals participate in the data collection, 9 of the participants were male and the rest were female. The data collection protocol was approved under the Rice University Institute Review Board (No. IRB-FY2018-434). The subjects were asked to sit in front of an RGB CMOS Camera (Blackfly BFLY-U3-23S6C). The video was recorded at 30 fps. For illumination, we used two dc LED arrays of total 500 lux and indoor ambient illumination. The laptop screen light also illuminated the participant’s face. Ground truth reference signal was simultaneously collected using the gold standard pulse oximeter CMS50D+ worn on the finger at a sampling rate of 60 Hz. The experimental set-up is shown in Fig. 4. The subjects had to perform five tasks, each for a duration of 2 min. The five tasks were as follows.

1. *Still*. Stay still facing the laptop in front of them. The subjects exhibited some facial movement.
2. *Reading*. Read a page of facts on the web browser. Subjects exhibited voluntary and involuntary facial motions.
3. *Watching*. Watch a scary movie trailer. Subjects showed expressions of fear or amusement.
4. *Talking*. Converse about any topic facing the laptop or camera.
5. *Deep breath*. Breathe normally for the first minute and take deep breaths at 6 breaths per minute for the second minute. The subjects inhale for 4 s and exhale for 6 s typically.

The dataset consisting of the raw unprocessed video frames at 30 fps and ground truth pulse oximeter signal is publicly available in Ref. 25.

### 3.2 Preprocessing of the Data

The data collected are videos focused on the participant’s face. We divide the two minute videos into two 60 s videos with an overlap of 20 s. We use existing iPPG algorithms as a preprocessing step to obtain the iPPG signal i(t) from the frames of the video. Face detection and tracking was the first step to account for the motion of subjects in front of the camera. We used a skin filter to detect the exposed skin area on the face and remove unwanted pixels that correspond to hair or glasses. Next, we divided the detected skin areas into four separate regions r, namely, left forehead, right forehead, left cheek, and right cheek and tracked it through the captured frames. To detect and track the shape of the face, we used OpenFace^26^ toolkit.

The next step involved extracting the iPPG signal from the four tracked regions. We averaged the pixel intensities for each region in each frame and each channel. The averaging reduces the effect of quantization noise. The 1800 samples (60s) time series computed for each region and each channel was detrended and filtered with an FIR bandpass filter of order 100 and a frequency range 0.5 to 5 Hz. The time series is the raw intensity variation signal. The measure intensity variation as a function of time, region, and channels are the following $i_{C}(r,t)$, where r corresponds to the four regions and C corresponds to R, G, B channels of the camera.

Recent work^15^ has shown that although the green channel has more PPG information than red and blue channels, the latter provide additional valuable information. Intensity signal measured from the red and blue channels can be used to eliminate illumination variation that occurs due to non-dc light source or motion. Hence, we adopted the prior method^15^ to compute the iPPG signal i(t). CHROM-RGB region wise iPPG signal s(r,t) was obtained using the chrominance method^15^ on the average region-wise pixel intensities of the three channels $i_{R}(r,t)$, $i_{G}(r,t)$, $i_{B}(r,t)$ separately shown in the following equation

$$x(r,t)=i_{R}(r,t)-2i_{G}(r,t),\;y(r,t)=1.5i_{R}(r,t)-i_{G}(r,t)+1.5i_{B}(r,t),\;s(r,t)=x(r,t)-\frac{\sigma [x(r,t)]}{\sigma [y(r,t)]}y(r,t),$$ (22)

where σ is the standard deviation of a signal.

In the above approach, the final iPPG signal is computed by a weighted averaging of the CHROM-RGB region-wise iPPG signal s(r,t). The four regions have different characteristics due to the microvasculature beneath the skin surface and the amount of motion exhibited during scenarios such as talking. Thus, factoring in the quality of the signal from each region improves the overall SNR of the final iPPG signal. In distancePPG, Kumar et al.^16^ showed that combining different parts of the face using the maximal ratio combination goodness metric $w_{\mathrm{MRC}}(r)$ shown in Eq. (23) improves the overall SNR of the signal.

The overall iPPG signal from the face $i_{\mathrm{CHROM}-\mathrm{MRC}}(t)$ is given by

$$i_{\mathrm{CHROM}-\mathrm{MRC}}(t)=\underset{r}{\sum }s(r,t)w_{\mathrm{MRC}}(r),\;\text{where}\;w_{\mathrm{MRC}}(r)=\frac{\int_{f_{\mathrm{hr}}-0.5}^{f_{\mathrm{hr}}+0.5},S(r,f)df}{\int_{0.5}^{5}S(r,f)df-\int_{f_{\mathrm{hr}}-0.5}^{f_{\mathrm{hr}}+0.5}S(r,f)df}.$$ (23)

In the above equation, S(r,f) is the normalized power spectral density of the signal s(r,t). The chrominance method^15^ and distancePPG^16^ were implemented with an epoch duration of 10 s with a 5-s overlap. The final iPPG signal $i_{\mathrm{CHROM}-\mathrm{MRC}}(t)$ is filtered with a FIR bandpass filter of order 200. We used the overlap-add method^15^ to stitch together the output iPPG signal from each epoch.

**Fig. 5:**
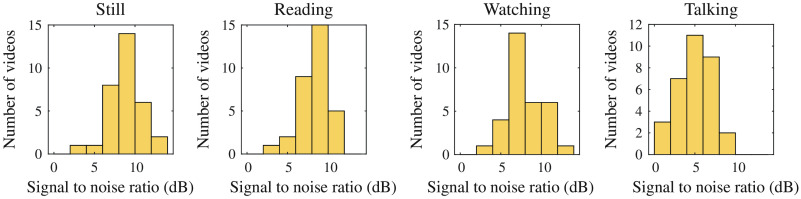
SNR of the iPPG signal for 60-s video computed with chrominance and distancePPG. Each scenario consists of 32 videos. The number of data point for low SNR regions is higher for the talking scenario.

$i_{\mathrm{CHROM}-\mathrm{MRC}}(t)$ was the final iPPG signal i(t) computed from the videos in the dataset. We had the ground truth PPG signal g(t) from the pulse oximeter. The signal-to-noise ratio (SNR) of the computed iPPG signal i(t) is given by

$$\begin{array}{c} \mathrm{SNR}=10\;\log[\frac{\int_{f_{\mathrm{hr}}-0.5}^{f_{\mathrm{hr}}+0.5}G(f)df}{\int_{f_{\mathrm{hr}}-0.5}^{f_{\mathrm{hr}}+0.5}|G(f)-I(f)|}], \end{array}$$ (24)

where G(f) and I(f) are the normalized power spectral density of the ground truth signal and the computed iPPG signal, respectively. The SNRs of the iPPG signals from the preprocessed videos are shown in Fig. 5.

## 4 Results

The performance of HRVCam was tested on the collected iPPG dataset. The main challenges in camera-based HRV estimation are to be able to capture HRV information accurately (a) across different skin tones and (b) different degrees of facial motion. Thus, we designed experiments to evaluate the algorithm’s robustness to skin tone and motion, respectively. We also assessed the algorithm’s performance at different autonomic states that capture low HRV and high HRV.

### 4.1 Prior Methods for Comparison

Prior works in HRV estimation from iPPG signals^7^^,^^16^^–^^18^^,^^19^ used a custom peak detection algorithm to identify beat timings and IBIs. Poh et al.^19^ used the NC-VT algorithm with 30% tolerance to filter computed IBIs to reduce error in the estimates. We implemented the NC-VT algorithm^14^^,^^19^ as one of the baseline methods for comparison. Our implementation consisted of two parts. The first part was the MATLAB^®^ findpeaks function that located the peak points of the iPPG signal i(t) with sufficient robustness. In the second part, the NC-VT filters that automatically discarded spurious estimates in IBI. We interpolated the iPPG signal to 500 Hz using spline interpolation before peak detection. The IBI were computed based on Eq. (3). In the subsequent sections, we refer to the implementation as Peak NC-VT. We also implemented the CDM method^10^^,^^11^ as a second baseline. The CDM method has only been evaluated on contact cPPG signals in prior works.^10^^,^^11^ We evaluated the CDM method on iPPG signals. The preprocessed iPPG signal i(t) is the input signal to the proposed estimator HRVCam and baseline comparison methods Peak NC-VT and CDM.

### 4.2 Evaluation Metrics

HRVCam and prior method estimate interbeat intervals from the iPPG signal computed from the 60 s video recording. RMSSD and SDNN metrics are computed from interbeat intervals (excluding first and last five IBIs due to filter edge effect). The computed metrics were validated against metrics calculated manually from the ground truth pulse oximeter signal. There is no widespread consensus regarding the metric used to quantify the error or the amount of error acceptable. We quantified the error using mean absolute error (mae) with standard deviation (sd) and Pearson correlation coefficient.

### 4.3 Robustness to Skin Tone

Table 1 summarizes the performance of the proposed method HRVCam and the baseline methods Peak NC-VT and CDM. The performance of HRVCam is comparable to prior method Peak NC-VT for light skin tones and the performance of HRVCam is better than Peak NC-VT for dark skin tones, as shown in Table 1. The error in the estimation with HRVCam arises because the algorithm utilizes instantaneous frequency from the first harmonic signal to measure HRV. In contrast, both Peak NC-VT and ground truth estimate HRV directly from the time difference between peaks of the signal. When the iPPG signal is of high signal quality, the peaks of the signal are correctly identified and match closely to the ground truth.

**Table 1:** Performance for different skin tones in the still scenario.

	SDNN		RMSSD	
	mae ± sd (ms)	Pearson’s correlation coefficient	mae ± sd (ms)	Pearson’s correlation coefficient
Light skin tone				
Peak NC-VT	3±3	0.96*	8±8	0.80*
CDM	6±5	0.89*	13±9	0.85*
HRVCam	3±4	0.92*	6±7	0.85*
Dark skin tone				
Peak NC-VT	3±3	0.98*	7±7	0.92*
CDM	4±3	0.96*	8±10	0.83*
HRVCam	2±1	0.99*	3±3	0.98*

* p-value<0.01

In low SNR, the peaks detected from a noisy iPPG signal do not match closely with the ground truth. Hence, the performance of the Peak NC-VT algorithm suffers HRVCam has a good performance in low SNR due to the DESA that handles the quantization noise. In Fig. 8(b), we show the artifacts present in the IBI estimates from Peak NC-VT. HRVCam shows an improvement for dark skin tones, as shown in Fig. 8(b).

**Fig. 6:**
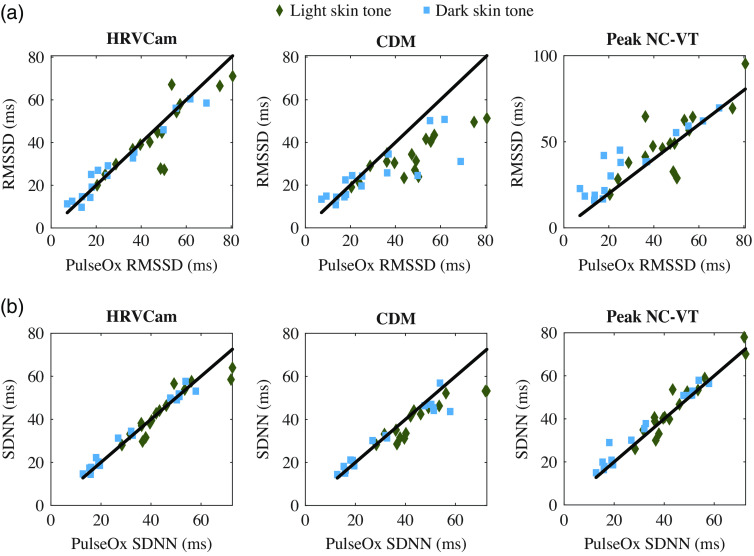
Scatterplot comparison of HRV metrics (a) RMSSD and (b) SDNN derived from iPPG with HRVCam, CDM, and Peak NC-VT methods, respectively, against ground truth pulse oximeter for different skin tones. Note that different y axis scales are used for the methods to accommodate all the points).

The CDM method displays significantly higher error than Peak NC-VT and HRVCam for the RMSSD metric. The cut-off frequency of the low-pass filter in the CDM method is defined as $f_{\mathrm{hr}}/3$.^11^ A cut-off frequency of $f_{\mathrm{hr}}/3$ leads to a narrower bandpass filter that filters out high-frequency HRV information. In Fig. 8, the IBIs estimated with the CDM method appear smoother than IBIs estimated from Peak NC-VT and HRVCam. Additional evidence is observed in Fig. 6(a) that shows that the RMSSD values estimated from CDM is lower than the ground truth pulse ox RMSSD values, especially for high RMSSD data points.

Note the SNR of the iPPG signal is slightly lower for darker skin tone than light skin tone. However, HRVCam provides lower error for darker skin tones. The reason is that the error in HRVCam estimate in the low-motion scenario arises due to assumptions in the algorithm and noise present in the signal. The error manifested due to the algorithmic assumptions depend on the HRV information contained in the signal. High HRV case faces more error than the low HRV case due to the loss of some high-frequency information while filtering the instantaneous frequency. The error due to algorithmic bias is higher in RMSSD as RMSSD captures high-frequency HRV information.

**Fig. 7:**
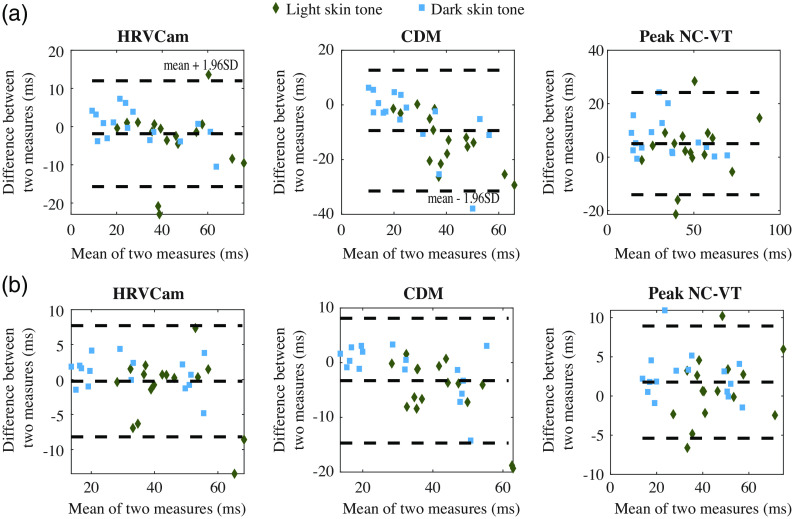
Bland–Altman plot of HRV metrics (a) RMSSD and (b) SDNN derived from iPPG with HRVCam, CDM, and Peak NC-VT methods, respectively, against ground truth pulse oximeter for different skin tones.

The Bland–Altman analysis for the still activity for RMSSD and SDNN is shown in Fig. 7. For RMSSD estimated using Peak NC-VT, the mean bias $\overset{¯}{d}=5\;\mathrm{ms}$ with 95% limit of agreement (mean bias 1.96 SD of the difference) is −14 to 24 ms. Using HRVCam reduces the error to $\overset{¯}{d}=2\;\mathrm{ms}$ with 95% limit of agreement of −16 to 12 ms. Using CDM $\overset{¯}{d}=-9\;\mathrm{ms}$ with 95% limit of agreement is −31 to 12 ms. For SDNN, Peak NC-VT delivers $\overset{¯}{d}=2\;\mathrm{ms}$ with 95% limit of agreement od −5 to 8 ms. HRVCam delivers $\bar{d}=-1\;\mathrm{ms}$ with 95% limit of agreement of −8 to 8 ms. CDM delivers $\overset{¯}{d}=-3\;\mathrm{ms}$ with 95% limit of agreement of −15 to 8 ms.

### 4.4 Robustness to Motion

The presence of facial movement degrades the SNR of the iPPG signal significantly. The amount of facial motion in reading a website, watching a video, and talking is different. Hence, we compared the performance of HRVCam and Peak NC-VT across all participants (light and dark skin tones) for the three different motion scenarios.

The reading activity had low facial movement across most participants with some sudden movements such as a smile or nod, which produced very short-duration motion artifacts. Although some peaks are corrupted by motion artifacts during the reading activity, the NC-VT filters out inaccurate IBI. Thus, the performances of HRVCam and Peak NC-VT are similar for the reading activity.

The mae ± sd for the RMSSD for the watching task was 11±15  ms for Peak NC-VT and 6±7  ms for HRVCam. The mae ± sd for the SDNN was 6±9  ms for Peak NC-VT and 3±3  ms for HRVCam. The mae ± sd for the RMSSD for the talking task was 63±78  ms for Peak NC-VT and 16±13  ms for HRVCam. The mae ± sd for the SDNN was 34±51  ms for Peak NC-VT and 11±13  ms for HRVCam. However, we observed 2 outliers (corresponding to 1 participant) data points compared to the other 30 (corresponding to 15 participants) data points. The outlier data points corresponded to a participant with a high amount of melanin pigment who exhibited a significantly higher amount of facial motion compared to other participants even during the watching task. Thus, the iPPG signal computed from the videos of this participant had very low SNR. If we excluded the data from that particular participant, we achieve a lower mae ± sd shown in Table 2 under watching and talking motion scenario. We have reported both values as it sheds light on the brittleness of the Peak NC-VT algorithm for low SNR signals. Overall, the HRVCam performance does not deteriorate drastically on the inclusion of the low SNR data points.

**Table 2:** Performance in different motion scenarios.

	SDNN		RMSSD	
Motion scenarios	mae ± sd (ms)	Pearson’s correlation coefficient	mae ± sd (ms)	Pearson’s correlation coefficient
Reading				
Peak NC-VT	3±2	0.99*	6±4	0.98*
CDM	5±6	0.92*	11±12	0.75*
HRVCam	3±3	0.97*	4±5	0.95*
Watching				
Peak NC-VT	5±7	0.92*	8±9	0.88*
CDM	6±5	0.94*	15±11	0.69*
HRVCam	2±2	0.99*	5±5	0.95*
Talking				
Peak NC-VT	28±45	—	53±70	—
CDM	16±32	—	24±29	—
HRVCam	9±8	0.82*	14±11	0.69*

* p-value<0.01

The amount of facial motion in the talking activity was higher than the watching activity. Thus, the performance of HRVCam was worse for talking scenarios. The performance of HRVCam is significantly better than Peak NC-VT and CDM for the talking scenario. In Fig. 9(b), we observe four data points with poor HRVCam performance. The iPPG signals corresponding to the four data points have an SNR of <5  dB. For the four data points, the motion interference power spectrum completely overlaps with the PPG power spectrum. Consequently, the adaptive filter cannot filter out the motion signal completely. The HRVCam estimates of the HRV parameters are inaccurate for the four data points.

The watching and talking activities introduce considerable motion interference in the iPPG signal. HRVCam performs significantly better (≥2x) than the Peak NC-VT algorithm. The Peak NC-VT algorithm fails to estimate the HRV metrics accurately because the peaks of the iPPG signal no longer capture the IBI in the presence of motion interference. HRVCam is designed to filter out the motion interference and so performs better in high-motion scenarios, as shown in Fig.

**Fig. 8:**
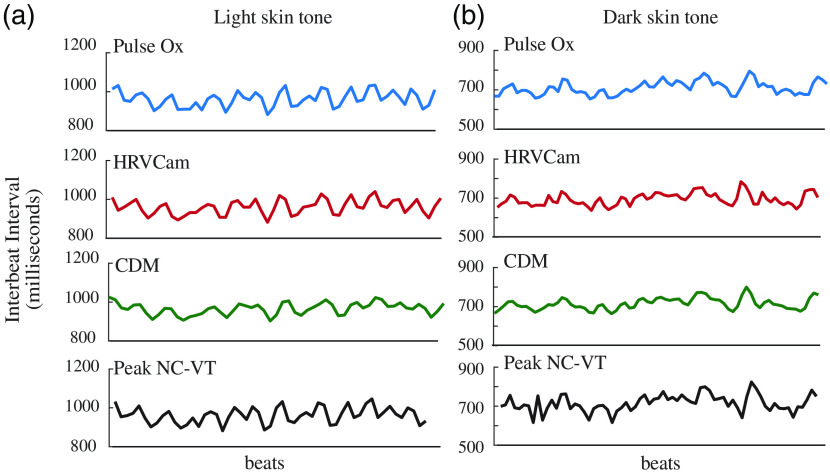
IBI estimates from HRVCam and Peak NC-VT against ground truth pulse oximeter (pulse ox) measurements for (a) light skin tone and (b) dark skin tone. The performance of HRVCam and Peak NC-VT is similar for light skin tone. However, for dark skin tone, IBI estimates from HRVCam follow ground truth more closely than Peak NC-VT. CDM estimates are smoother than HRVCam and Peak NC-VT.

11.

**Fig. 9:**
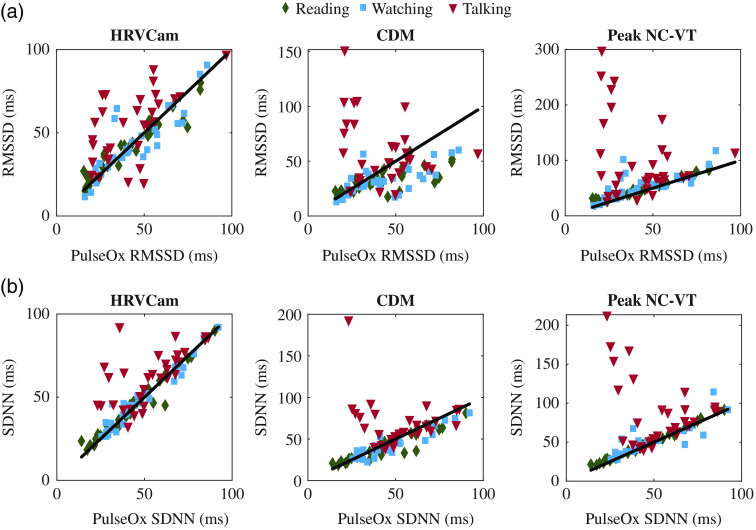
Scatterplot comparison of HRV metrics (a) RMSSD and (b) SDNN derived from iPPG with HRVCam, CDM, and Peak NC-VT methods, respectively, against ground truth pulse oximeter for different motion scenarios. Note that different y axis scales are used for the methods to accommodate all the points).

**Fig. 10:**
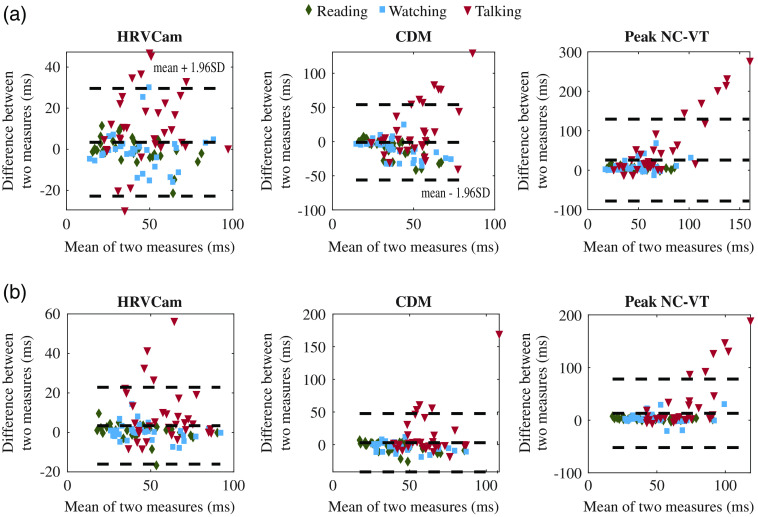
Bland–Altman plot of HRV metrics (a) RMSSD and (b) SDNN derived from iPPG with HRVCam, CDM, and Peak NC-VT methods, respectively, against ground truth pulse oximeter for different motion scenarios.

The Bland–Altman analysis for motion activities for RMSSD is and SDNN is shown in Fig. 10. For RMSSD estimated using Peak NC-VT, the mean bias $\overset{¯}{d}=26\;\mathrm{ms}$ with 95% limit of agreement (mean bias 1.96 SD of the difference) of −78 to 130 ms. Using HRVCam reduces the error to $\overset{¯}{d}=3\;\mathrm{ms}$ with 95% limit of agreement of −22 to 30 ms. Using CDM $\overset{¯}{d}=-1\;\mathrm{ms}$ with 95% limit of agreement of −56 to 54 ms. For SDNN, Peak NC-VT delivers $\overset{¯}{d}=13\;\mathrm{ms}$ with 95% limit of agreement of −52 to 78 ms. HRVCam delivers $\overset{¯}{d}=3\;\mathrm{ms}$ with 95% limit of agreement of −16 to 23 ms. CDM delivers $\overset{¯}{d}=3\;\mathrm{ms}$ with 95% limit of agreement of −41 to 47 ms.

**Fig. 11:**
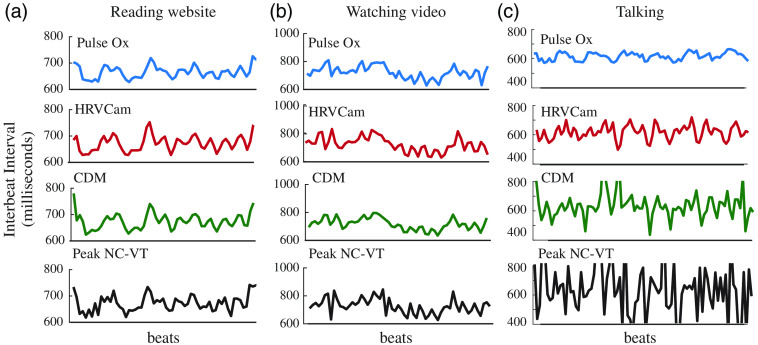
IBI estimates from HRVCam, Peak NC-VT, CDM, and ground truth pulse oximeter for different motion scenarios (a) reading a website, (b) watching videos, and (c) talking. HRVCam follows ground truth pulse oximeter closely in reading and watching scenarios. In talking, the performance of HRVCam suffers. However, HRVCam provides significantly better estimates than Peak NC-VT and CDM.

**Fig. 12:**
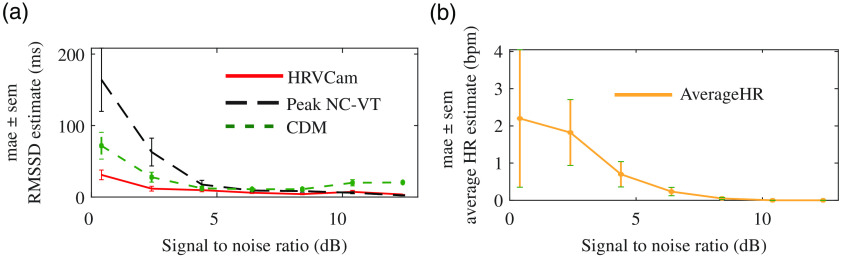
(a) Distribution of mae in RMSSD across different SNR obtained from iPPG signal computed for different activities. For lower SNR [calculated with Eq. (24)] videos, HRVCam shows large improvements. (b) Distribution of error in average heart rate across SNR

Overall, the mae for HRVCam RMSSD estimate is consistently lower compared to Peak NC-VT across a range of SNR, as shown in Fig. 12(a). The performance of CDM improves over Peak NC-VT because the low-pass filter filters out part of the motion interference. However, it does not completely filter out the motion interference in most cases, leading to inaccurate HRV estimates. In Fig. 12(b), we present the average heart rate estimated by detecting the peak frequency in the power spectral density of the iPPG signal. The average heart rate estimated is within three beats per minute.

### 4.5 Sensitivity of HRVCam

HRVCam significantly increases the accuracy of HRV estimation in iPPG systems. We conducted a deep breathing activity to evaluate if HRVCam is sensitive enough to differentiate autonomic states. In this activity, the participant first breathes normally for 1 min. Then the participant performs deep breathing at a rate of ∼6 breaths per minute for the second minute. The deep breathing activity causes the IBIs to synchronize with the breathing pattern due to the influence of the autonomic nervous system regulation. As a result, the RMSSD parameter significantly increases during the deep breathing activity.

In our experiment, we first examined if there was a significant increase in RMSSD due to deep breathing with the ground truth estimates. The difference between ground truth HRV during normal breathing and deep breathing was significant with Wilcoxon signed-rank test parameter W=6. The critical value for W at N=16 (p≤0.01) is 19. Thus, the deep breathing activity indeed led to higher values of RMSSD metric.

Pearson’s correlation coefficient of HRVCam estimates with ground truth HRV in the normal state is 0.90, p≤0.01, and the correlation during deep breathing is 0.94, p≤0.01. The corresponding coefficient of correlation for Peak NC-VT is 0.88, p≤0.01, and 0.81, p≤0.01. The coefficient of correlation for CDM is 0.6, p≤0.01, and 0.54, p≤0.01.

The coefficient of correlation between the changes in HRV estimated by HRVCam with the ground truth change in HRV is 0.86 (p<0.01). In contrast, the corresponding number for Peak NC-VT is 0.71 (p<0.01). The coefficient of correlation for CDM is 0.38.

**Fig. 13:**
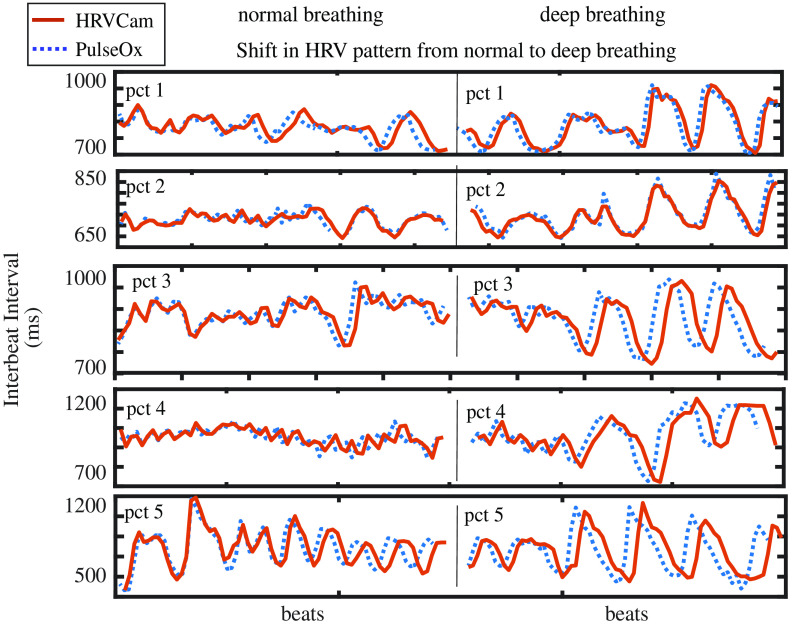
Change in HRV pattern from normal breathing to deep breathing in a participant: In the left panel, the pattern of IBI is random while the person breathes normally, and its HRV variability is lower. On the right panel, during deep breathing due to respiration sinus arrhythmia, the pattern of the IBI is sinusoidal syncing with the respiration, and HRV variability increases. We show examples of five random participants (top to down).

In Fig. 13, the IBIs obtained from HRVCam carefully follow the ground truth observation from the pulse oximeter. The HRV signal is distinctly different for two states, and HRVCam estimates the signal accurately.

## 5 Discussion and Analysis

### 5.1 Effect of SNR on HRV Estimation

**Fig. 14:**
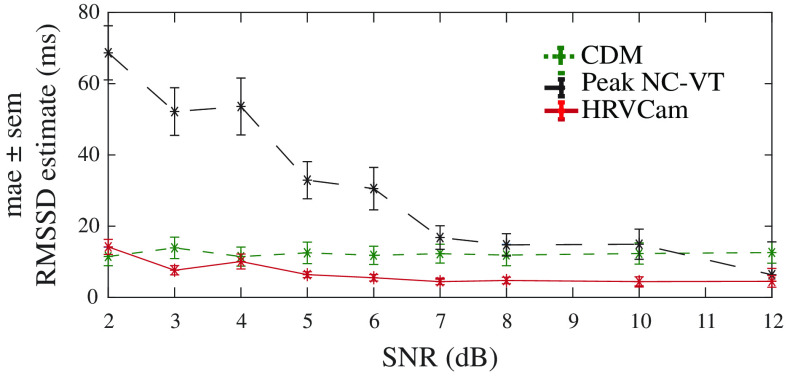
The SNR of the cPPG signal degrades on increasing the power of additive white noise added to the signal. The performance of peak-based approach (Peak NC-VT) deteriorates at a faster rate than frequency-based approaches (HRVCam and CDM).

The estimate of HRV metrics is very sensitive to signal quality. We add additive white Gaussian noise to clean ground truth cPPG signals from multiple participants to simulate various SNR regimes. We show with simulation in Fig. 14 that the performance of HRVCam degrades in the low SNR regime of the contact pulse signal (cPPG).

The overall improvement in the error of HRV metrics due to HRVCam over prior peak detection-based approaches is consistent over the range of low SNR values. HRVCam is inspired from CDM.^10^^,^^11^ Hence, for cPPG signal corrupted with additive white noise, the performance of HRVCam is comparable to previous PFDM approaches.^10^^,^^11^ However, HRVCam performs better than CDM for iPPG signals in the low SNR regime shown in Fig. 12(a). The low SNR of iPPG signals arise from the presence of motion artifacts. Therefore, in the presence of motion artifacts in the iPPG signals, the superior performance of HRVCam is due to the adaptive bandpass filter.

In this work, we have combined state-of-the-art robust iPPG estimators’ chrominance-based rPPG algorithm^15^ and distancePPG algorithm^16^ to achieve the highest possible SNR for the iPPG signal in any given scenario. The reason for using robust iPPG estimators was to fairly evaluate the performance of Peak NC-VT because peak based approaches has high performance in the high SNR regime.

### 5.2 Adaptive Bandpass Filter Bandwidth

The adaptive filter’s bandwidth aBW is designed to filter out high-motion interference within the HRV band of interest. The filter’s bandwidth would be narrower to reject motion interference while retaining HRV information. Thus, the filter bandwidth should be narrower in scenarios of high motion. The filter bandwidth is an indirect indicator of the signal quality of the iPPG signal.

**Fig. 15:**
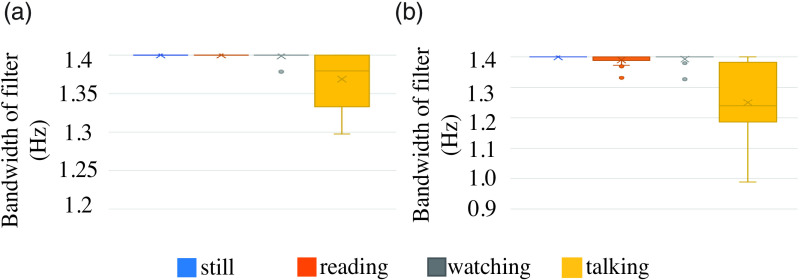
Change in adaptive filter bandwidth for different scenarios: the range of motion increases from still task to talking. With an increase of motion, the motion interference component in the iPPG signal increases and the bandwidth of the bandpass filter is the largest for the still task and smallest for talking task.

Figure 15 shows the distribution of the estimated bandwidth. We validate our procedure of estimation of the bandwidth and check if the distribution differs as expected for low- and high-noise scenarios. A single data point in Fig. 15 refers to the mean bandwidth of the adaptive filter for the iPPG signal of a video. The mean bandwidth is calculated as the average bandwidth across the epochs from a single video. The adaptive bandpass filter bandwidth depends on the frequency and magnitude of the motion interference signal present in the iPPG signal. The frequency and magnitude of motion interference signal depend on the intensity, type of facial motion the participant exhibits, and the CHROM method’s performance in suppressing motion interference during preprocessing. We observed that in the still, reading, and watching scenarios, the performance of the CHROM method was not significantly different across skin tones. Hence, the filter bandwidth depended on the amount of motion exhibited by the participants irrespective of their skin tone. However, in the talking scenario, the performance of the CHROM method was significantly worse for very dark skin tone participants compared to light skin tone participants despite both exhibiting similar facial motion during the talking activity. For the very dark skin tone participants, the filter’s bandwidth was narrow to filter out the strong motion interference present in the signal.

The filter design was constructed to accommodate iPPG ranging from very low-quality to high-quality SNR. The filter is designed to retain HRV information and reject considerable motion interference if present. However, the parameters of the filter can be specified separately for each activity. If a potential application consists purely of one type of activity such as talking, setting a narrower initial bandwidth for the filter would be beneficial for such an application.

### 5.3 Frequency Domain HRV Parameters

In our work, we limited our analysis to time-domain HRV metrics such as RMSSD and SDNN because of the time duration of our dataset was limited to 120 s. We did not report frequency domain parameters as a duration longer than 120 s is needed for reliable calculation of low-frequency and high-frequency HRV metrics.^1^^,^^27^^–^^29^ As it was difficult to maintain specific experimental conditions for a longer period of time without participant discomfort, we used only continuous 120-s time duration for the data collection. As a future direction, longer time duration videos need to be collected efficiently to evaluate frequency-domain HRV parameters. The participants’ discomfort arose mainly from performing a single-activity continuously while keeping the hand attached to the finger pulse oximeter very still to ensure high quality of ground truth data. We can imagine two modifications to the experimental protocol to enable the collection of longer videos. First would be the use of a comfortable and robust contact device for capturing ground truth PPG signal. The second would be to ask participants to work on their laptops normally while being recorded by the camera. We would then manually label the different activities and resultant degree of motion in the videos by visual inspection.

## 6 Conclusion

We propose an estimator HRVCam that can robustly extract HRV signals from noisy iPPG signals based on adaptive bandwidth filter pulse frequency demodulation. We quantified the accuracy of HRV parameters with existing iPPG algorithms under different application-oriented scenarios such as reading, talking, and watching videos for both light and dark skin tones individuals.

HRVCam improves the accuracy of estimated time-domain HRV metrics. We validated the different steps of the algorithm against the ground truth obtained by an FDA approved pulse oximeter. We showed that the correlation coefficient between HRVCam estimates and ground truth estimated for both light and dark skin tone subjects under different scenarios was higher than the state-of-the art peak detection approaches. As the required accuracy is not well defined for HRV parameters, we tested the sensitivity of HRVCam using the deep breathing test. HRVCam could estimate the change in HRV pattern from normal to deep breathing states with a correlation coefficient of 0.9 (p<0.01). We hope that the thorough analysis conducted in this work and the proposed HRVCam algorithm will boost confidence in the application of noncontact camera-based HRV monitoring.

Our future work includes open-sourcing the code for broader use in research projects. We have already made the dataset collected public. A future extension would be to implement HRVCam into a mobile application that can enable the translation of HRVCam for real-time camera HRV applications.

## Code, Data, and Materials Availability

The dataset collected in this study is publicly available in the raw format in Ref. 25.
